# Efficacy Analysis of Day Surgery A1 Pulley Release for Pediatric Trigger Thumb

**DOI:** 10.3389/fped.2021.734115

**Published:** 2021-09-14

**Authors:** Yan Linhua, Jiang Linjun, Qu Xiangyang, Liu Xing, Li Ming, Wu Jun

**Affiliations:** Department of Orthopedics, Children's Hospital of Chongqing Medical University, Ministry of Education Key Laboratory of Child Development and Disorders, Chongqing Key Laboratory of Pediatrics, National Clinical Research Center for Child Health and Disorders, Chongqing, China

**Keywords:** children, patient satisfaction, pulley release, surgical complications, thumb

## Abstract

**Objective:** To investigate clinical application of day surgery A1 pulley release for pediatric trigger thumb.

**Methods:** We retrospectively analyzed the clinical data of 1,642 children with trigger thumb who were treated with day surgery A1 pulley release at our hospital, including satisfaction surveys, functional recovery, and complications.

**Results:** The operative time for unilateral and bilateral tenolysis was 4.8 ± 3.1 and 9.2 ± 3.8 min, respectively. Three children had postoperative fever and were discharged on the 2nd day after surgery. The rest of the children were discharged on the day of surgery. All incisions healed primarily, and no complications of vascular and nerve injury were reported. The patients' degree of satisfaction with the medical treatment process, diagnosis and treatment workflow, treatment effectiveness, length of hospital stay and hospitalization cost, and discharge guidance were 97.9, 96.1, 99.3, 91.1, and 98.5%, respectively. The follow-up period was between 5 months and 3 years and 1 month. Four children experienced symptom relapse after the operation, and re-tenolysis was performed in one of them. At the final follow-up, the appearance and function of the thumb had recovered well in all cases.

**Conclusion:** Day surgery A1 pulley release can effectively release tendon sheaths and has a short operative time, no complications of vascular and nerve injury, and good recovery of thumb function. It is a safe and reliable procedure with high patient satisfaction, and it is worthy of clinical promotion.

## Introduction

Trigger thumb is a commonly encountered disease in pediatric practice, accounting for 87–93% of finger-stenosing tenosynovitis. It is most common in children aged 1–4 years, with an incidence of 1–3% (1). The main pathological changes are collagen degeneration of the flexor pollicis longus muscle tendon and thickening and narrowing of the tendon sheath (2). To date, non-surgical and surgical treatments for pediatric trigger thumbs are available (3, 4). Non-surgical treatments include local hot compress, massage, medicine fumigation, and intrathecal injection of medicine (5). The success rate of non-surgical treatment may vary, and the treatment duration is long (6). Open A1 pulley release is currently considered to be a safe and effective treatment for pediatric trigger thumb (7).

Day surgery refers to a procedure that requires the participation of an anesthesiologist and the completion of admission, operation, and discharge in a single working day (8). A large number of clinical studies have shown that the day surgery model has the advantages of safety and efficiency. Day surgery is safer than outpatient surgery (9, 10). Compared with traditional inpatient surgery, day surgery can effectively shorten the length of hospital stay, reduce hospitalization costs, and optimize the allocation of medical resources (11). In developed countries such as Europe and the US, most common surgical indications are treated with day surgery, which accounts for ~40–70% of all surgeries (12). In the 1960's, day surgery for indirect inguinal hernia in children was performed in China (13). With the development of medical technology and the continuous advancement of anesthesia technology, the types of diseases indicated for day surgery and the amount of day surgeries performed in the field of pediatric surgery have increased yearly (14). Nonetheless, day surgery for pediatric trigger thumb has not been reported.

Day surgery has been performed since 2014 at the Children's Hospital of Chongqing Medical University, China. With the aim of ensuring the safety of clinical medical care and the continuous improvement of the medical management system and standards, we improved the day medical services workflow and gradually created a day medical service system for pediatric patients (15). This article retrospectively analyzes the clinical data of 1,642 children who underwent tenolysis of the thumb under the day surgery model at our hospital and explores the clinical experience of day surgery A1 pulley release for the treatment of pediatric trigger thumb.

## Methods

### Study Subjects

We retrospectively analyzed the clinical data of children who underwent day surgery tenolysis of the thumb at Children's Hospital of Chongqing Medical University from April 2014 to December 2019. Patients who were lost to follow-up or had incomplete clinical data were excluded. A total of 1,642 children were included in this study, including 644 males and 998 females. Their average age was 3.17 ± 1.92 years (range: 1 year 3 months to 8 years 1 month). From all the subjects, 1,381 patients were under 3 years old, 194 patients were between 3 and 6 years old, and 67 were over 6 years old. Unilateral trigger thumb was diagnosed in 1,354 patients (on the left side in 604 patients and on the right side in 750 patients), and bilateral trigger thumb was diagnosed in 288 patients. The general data of the children is shown in Table 1.

**Table 1:** Demographics of the pediatric patients.

		**Numbers (n)**	**Ratio (%)**
**Age**			
	≤ 3 years	1,381	84.1
	>3, ≤ 6 years	194	11.8
	>6 years	67	4.1
**Sex**			
	Male	644	39.2
	Female	998	60.8
**Hand involvement**			
	Left	604	36.8
	Right	750	45.7
	Biolateral	288	17.5
Stage of triggers^*^			
	Stage I (tumor type-pretriggering)	0	0
	Stage II (active triggering)	135	8.2
	Stage III (passive triggering)	281	17.1
	Stage IV (rigid type-contracture)	1,226	74.7

* *According to modified Green DP classification*.

### Diagnostic and Treatment Workflow for Day Surgery

**Figure 1:**
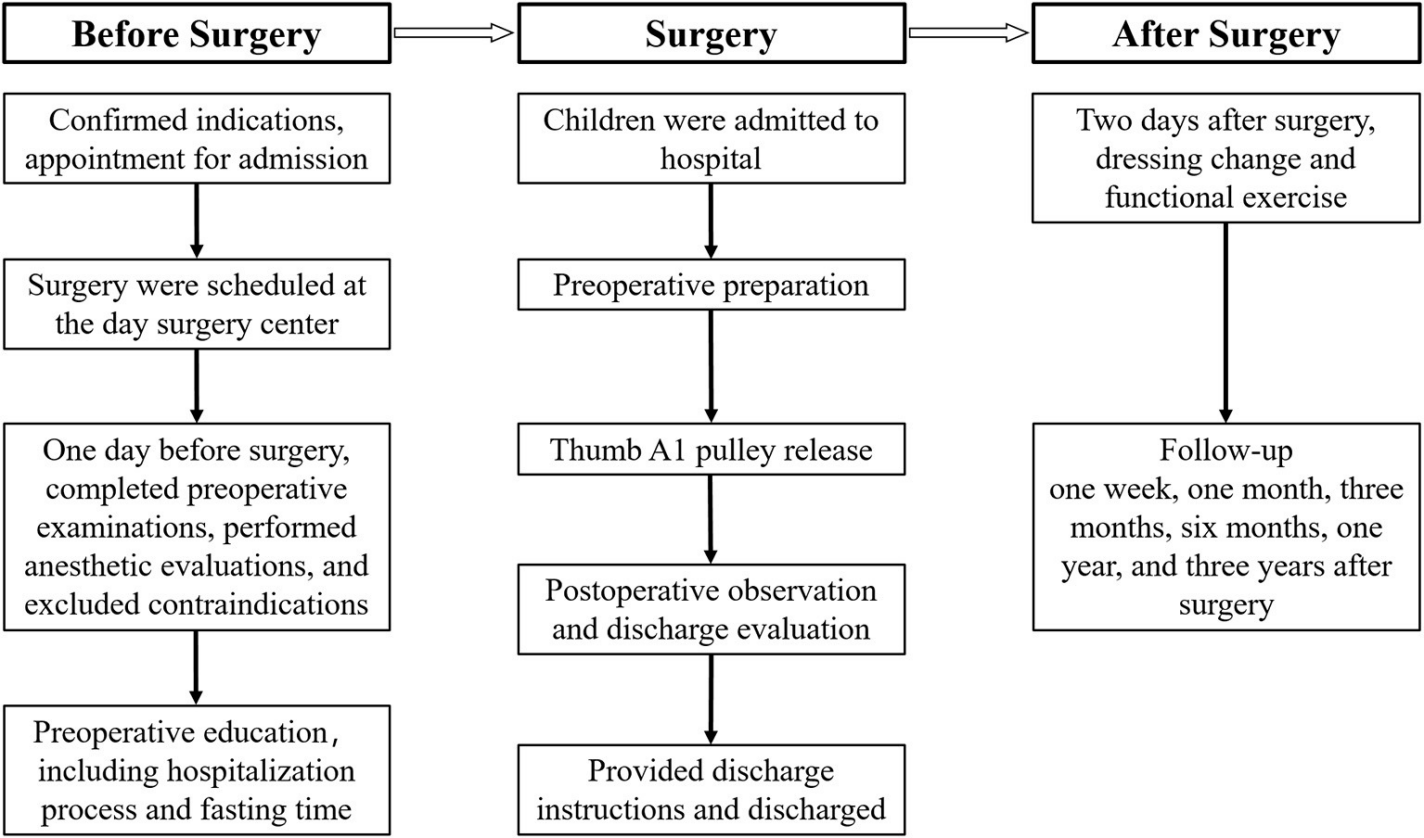
Diagnostic and treatment workflow for day surgery.

The diagnostic and treatment workflow for day surgery in our hospital is shown in Figure 1. The thumb A1 pulley release is performed. Vital signs, the incision condition and the blood supply of the fingertip are observed after the surgery. The child is evaluated for discharge. The dressing is changed 2 days after the operation, and finger function training begins. The child is followed up at the Pediatric Orthopedic Specialty Clinic regularly after the surgery.

### Surgical Procedure

After successful intravenous induction of anesthesia, median nerve block anesthesia is performed under ultrasound guidance (16). An 0.5–1.0-cm incision is made along the transverse lines at Notta's nodule on the palm side of the metacarpophalangeal joint to incise the skin layer by layer and separate the subcutaneous tissue. Attention is paid not to injure the blood vessels and nerves. The flaps and neurovascular tissue are retracted to opposite sides to expose the flexor pollicis longus muscle tendon sheath, and the fibrous layer of the hyperplastic tendon sheath is incised along the longitudinal axis of the tendon sheath. The synovial layer is not opened. After pulley release, the ROM of the interphalangeal joint of the thumb is examined. If the ROM is normal, with smooth motion of the tendon, and the tendon is not entrapped, the incision is closed. The incision is dressed, and the thumb is immobilized in the dorsal extension position (open position of the thumb-index web space) (17).

### Postoperative Observation and Discharge Evaluation

The vital signs and incision condition are observed carefully after the operation. The discharge criteria are based on the Aldrete and Marshall Chung scoring system (18) and are as follows: ① Vital signs are stable for more than 2 h; ② The child can drink water and urinate; ③ No frequent nausea and vomiting; ④ Good orientation ability; ⑤ No bleeding in the incision and good blood supply at the fingertips.

With reference to relevant domestic and foreign literature regarding day surgery and based on the 8-dimensional Hospital Consumer Assessment of Healthcare Providers and Systems (HCAHPS) scale covering the core experience of patients during hospitalization (19), the “Child Satisfaction Questionnaire” was designed. The satisfaction evaluation includes satisfaction with the medical treatment process, treatment effectiveness, service of the medical staff, and discharge guidance. Before a child is discharged from the hospital, the surveyors distribute and retrieve this questionnaire at bedside.

## Results

The wait time for day surgery was 1–13 days in pediatric patients. The operative time for unilateral or bilateral injury was 4.8 ± 3.1 min and 9.2 ± 3.8, respectively. After the release of the tendon sheath, unrestricted passive movement of the interphalangeal joint of the thumb was observed. No complications of vascular or nerve injury occurred during or after the operation. None of the children had frequent nausea, vomiting, severe pain, and no serious complications, such as death, occurred after the operation. Three children developed postoperative fever and were transferred to a specialty ward. After symptomatic treatment, they were discharged the next day. All of the children were discharged on the day of surgery except for the three who had delayed discharge. All incisions were free of infection and healed primarily. The sutures were removed 12–14 days after surgery.

In terms of the patients' satisfaction with the service of the medical staff, the satisfaction with the doctor was 97.8%, the satisfaction with the nursing staff and anesthesiologist was 99.6%, and the satisfaction with the laboratory service staff and the operating room staff was 96.2%. The patients' satisfaction with the medical treatment process, diagnostic and treatment workflow, treatment effectiveness, length of hospital stay and hospitalization cost, and discharge guidance were 97.9, 96.1, 99.3, 91.1, and 98.5%, respectively (Table 2).

**Table 2:** Patient satisfaction [n(%)].

	**Very Satisfied**	**Satisfied**	**Somewhat satisfied**	**Dissatisfied**
Environment of the medical facility	704 (42.9)	713 (43.4)	190 (11.6)	35 (2.1)
Diagnostic and treatment workflow	594 (36.2)	698 (42.5)	286 (17.4)	64 (3.9)
Treatment effectiveness	553 (33.7)	880 (53.6)	197 (12.0)	12 (0.7)
Length of hospital stay and expenses	682 (41.5)	642 (39.1)	172 (10.5)	146 (8.9)
Discharge guidance	499 (30.4)	965 (58.8)	153 (9.3)	25 (1.5)

**Figure 2:**
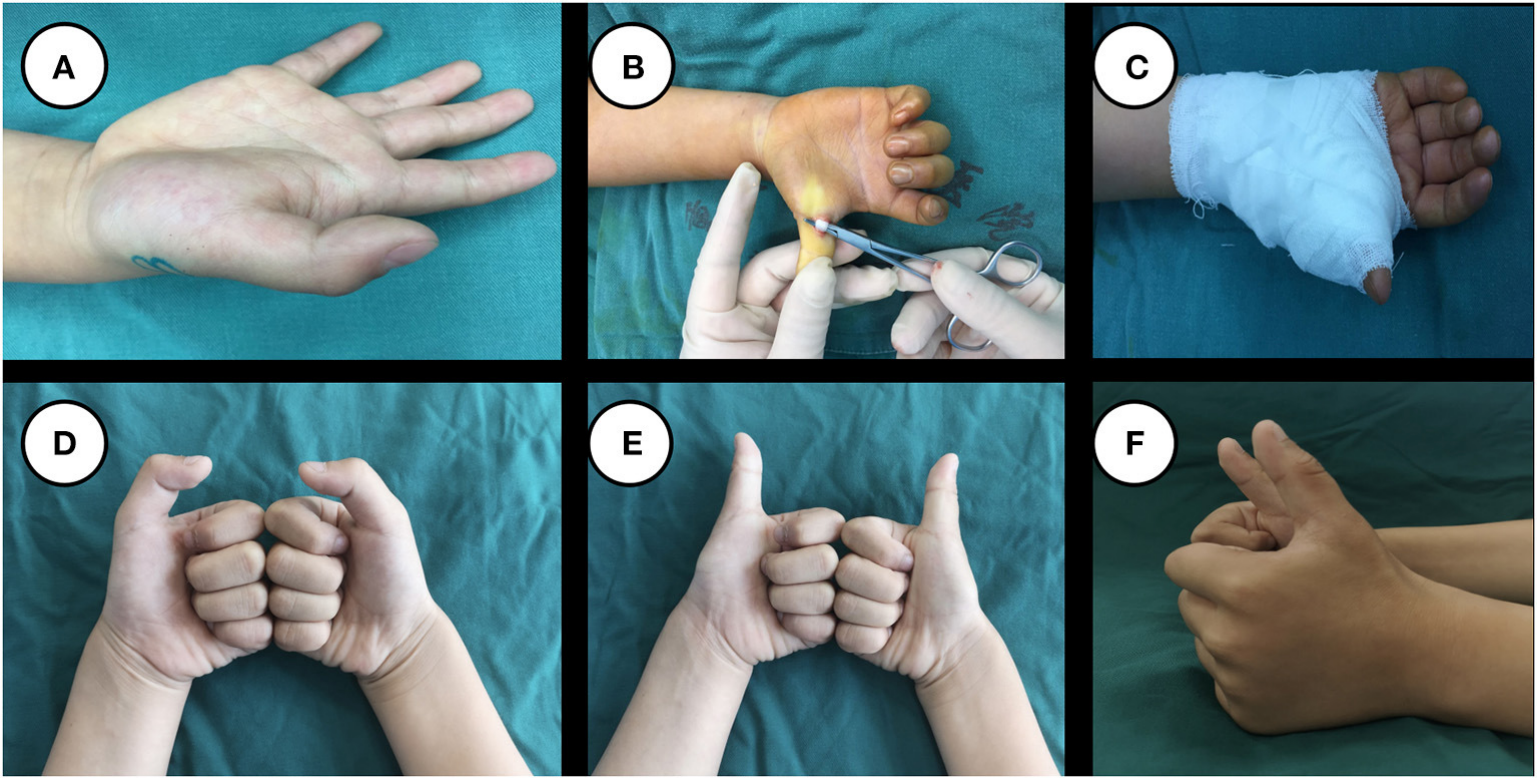
A 3-year and 5-month-old boy presented with inability to extend his right thumb for 4 months. Before the operation, extension of the interphalangeal joint of the thumb was limited **(A)**. A1 pulley release was performed. After the tendon sheath was released, the motion of the interphalangeal joint of the thumb returned. The tendon glided well without entrapment and with a normal ROM **(B)**. The incision was closed with sutures, and the thumb was immobilized in the dorsal extension position **(C)**. A follow-up visit at 1 year and 9 months after the operation showed a well-healed incision, no obvious scar formation, normal appearance of the thumb, and unrestricted movement of the interphalangeal joint of the thumb, frontal view of the thumb in flexion **(D)**, frontal view of the thumb in straight position **(E)** and side view of the thumb in straight position **(F)**.

The follow-up period was ranged from 5 months to 3 years and 1 month. Relapse occurred in 4 children after the operation. Repeat tenolysis was performed in one of them due to incomplete release. The other 3 patients were cured after conservative treatment, and their recurrence might be related to postoperative tendon adhesion. At the final follow-up, the Notta's nodules at the metacarpophalangeal joints disappeared in 1,341 cases but were still palpable in 301 cases. There was no significant difference in the range of flexion and extension of the interphalangeal joint of the affected thumb compared with that of the healthy thumb, and the thumb had good functions of abduction, adduction, and thumb opposition. The appearance of the thumb was normal, and no sympathetic muscle atrophy occurred in the hand. A typical case is shown in Figure 2.

## Discussion

The following principles apply to diseases indicated for pediatric day surgery: (1) The disease is common in children, the identical procedure is performed in a number of patients, and the operation can be implemented according to clinical pathway specifications; (2) the surgical technique is mature and minimally invasive, with a short operative time; and (3) the procedures are associated with mild postoperative pain, quick recovery, few complications, and no requirements for special care (20). Han et al. used A1 pulley release to treat trigger thumb in children and achieved good outcome of complete tendon release and well-recovered joint function, without postoperative pain or complications such as vascular or nerve damage (21). Dinham et al. reported the use of A1 pulley release to treat 105 patients with trigger thumb (131 thumbs). The range of motion (ROM) of the interphalangeal joints of the thumb was recovered completely in 100 thumbs. Reoperation was required in one thumb due to incomplete release. Incision infection was reported in one patient. Three patients had more than 15° flexion deformity of the interphalangeal joints for unknown reasons. The surgical remission rate was 95.2% (22). McAdams et al. retrospectively analyzed the clinical data of 21 patients with trigger thumb (30 thumbs) with an average of 15 years of follow-up and reported no recurrence or interdigital dysfunction. They concluded that A1 pulley release is an effective treatment for pediatric trigger thumb (23). In this study, 1,642 patients with thumb-stenosing tenosynovitis (1,930 thumbs) were included. The operative time was only a few minutes, and no complications such as vascular or nerve damage or severe pain after the operation were reported. This study shows that as a procedure for the treatment of pediatric trigger thumb, A1 pulley release is appropriate for day surgery.

The quality and safety of day surgery have become important factors in its development. Therefore, day surgery must implement a perioperative management system that is same as that used in traditional inpatient surgery (12, 13). Ma et al. analyzed the clinical data of 129,869 pediatric patients undergoing day surgery under the centralized admission management model and found that strict adherence to the “three requirements” and “three evaluations” standards, a reasonable hospital observation time, standardized discharge education and follow-up, and a sound day surgery safety system can effectively reduce the rates of delayed discharge and postoperative complications. Therefore, the quality, safety, and overall management of day surgery are the same as or even higher than those of traditional surgery (14). In this study, day surgery A1 pulley release for pediatric trigger thumb was selected as the research object. The patient admission and chief surgeon privilege systems were strictly implemented, and a standardized preoperative evaluation, anesthesia evaluation, and discharge evaluation were carried out. Postoperative follow-up showed complete release of the tendon sheath, well-recovered thumb function, and no complications such as vascular or nerve injury. The results suggest that day surgery A1 pulley release for pediatric trigger thumb is a safe and reliable procedure.

In this study, the wait time for scheduled surgery was between 1 and 13 days. Three children had postoperative fever and were discharged on the 2nd day after surgery. The other children were discharged on the day of surgery. The patient satisfaction survey showed a high degree of satisfaction with the medical treatment process, diagnostic and treatment workflow, treatment effectiveness, length of hospital stay, hospitalization cost, and discharge guidance.

## Conclusion

A1 pulley release is a procedure for the treatment of pediatric trigger thumb that offers a short operative time, minimal trauma, quick recovery, and reduced complications. It is appropriate for completion as a day surgery. Standardized perioperative management is an important guarantee of the safety and quality of day surgery. Reasonable surgical workflow management provides considerable support for the promotion of day surgery. Day surgery A1 pulley release can effectively release tendon sheaths, with good recovery of thumb function and no complications, such as vascular nerve damage. It is safe and reliable procedure with high patient satisfaction, and it is worthy of clinical promotion.

## Data Availability Statement

The raw data supporting the conclusions of this article will be made available by the authors, without undue reservation.

## Ethics Statement

The studies involving human participants were reviewed and approved by Children's Hospital of Chongqing Medical University. Written informed consent to participate in this study was provided by the participants' legal guardian/next of kin. Written informed consent was obtained from the individual(s) legal guardian/next of kin for the publication of any potentially identifiable images or data included in this article.

## Author Contributions

WJ, QX, LX, and LM were involved in the design of the project and participated in the surgery and follow-up. YL and JL participated in the preoperative preparation and discharge education. YL, JL, and QX collected the data and conducted the analysis. WJ, YL, and QX drafted the manuscript. LX and LM made the critical revisions. All of the authors read and approved the final manuscript.

## Funding

This work was supported by the Children's Hospital of Chongqing Medical University (YBXM-2019-10).

## Conflict of Interest

The authors declare that the research was conducted in the absence of any commercial or financial relationships that could be construed as a potential conflict of interest.

## Publisher's Note

All claims expressed in this article are solely those of the authors and do not necessarily represent those of their affiliated organizations, or those of the publisher, the editors and the reviewers. Any product that may be evaluated in this article, or claim that may be made by its manufacturer, is not guaranteed or endorsed by the publisher.
